# Spotlight on sensors as tools for biointelligence

**DOI:** 10.1007/s00216-026-06332-5

**Published:** 2026-02-03

**Authors:** Günter Gauglitz

**Affiliations:** Institute for Theoretical and Physical Chemistry, Eberhard-Karls-University, Tübingen, Germany

At Euroanalysis 2025 in Barcelona, a lecture titled *“Sensors – Tools for Biointelligence?”* was held on the occasion of the  DAC-EuChemS Award ceremony. What does the term *biointelligence* mean, where does it come from, and does *biointelligence* require analytics or sensors in particular? This helps assess whether sensors are suitable tools and perhaps confirm this with applications.

*Biointelligence* is a relatively new interdisciplinary concept that has emerged from the convergence of life sciences, engineering, and information technology to create sustainable, adaptive, and intelligent systems. These systems integrate biological components (e.g., microorganisms, cells), technical artifacts, and digital control to enable new forms of value creation [1]. A biointelligent system efficiently and sustainably uses biological resources, contains biological and technical components, seeks feedback and interaction with its environment, and often exhibits features of artificial intelligence. The term was developed around 2017 during the BIOTRAIN preliminary study by the Fraunhofer Institute for Manufacturing Engineering and Automation (IPA) [2]. The goal was to analyze the biological transformation of industrial value creation and define new innovation paths. This transformation occurs in three stages: (1) Bioinspiration – transfer of biological principles (e.g., bionics), (2) Biointegration – embedding biological components into technical systems, (3) Biointeraction – emergence of biointelligent systems through feedback and networking [1]. The term was shaped by an interdisciplinary team, notably Prof. Dr.-Ing. Thomas Bauernhansl, Dr. Robert Miehe, and Yannick Baumgarten (Fraunhofer IPA). History, concept, and biointelligent systems have recently been discussed [3]. A scientifically sound analysis of the current research status and future perspectives of *biointelligence* complements the Fraunhofer studies with: (1) a critical review, (2) a categorization of biointelligent systems, (3) a reflection on societal and ethical implications [4].

The term *biointelligence* was first mentioned in the late twentieth century, described as the “true intelligence of nature” in contrast to machine intelligence [5]. Early uses of the term appear in contexts such as bioinformatics, systems biology, and robot-assisted surgery [6]. Other works address the integration of bioinformatics and systems biology into medical decision-making [7]. These sources show that in earlier contexts, the term “*biointelligence*” was often understood as the intelligent use of biological data and systems—for example, to control surgical robots or to model complex biological processes in cancer research. Next, the “Biointelligence Competence Center e.V.” was founded in 2021 as an open network organization aiming to further develop *biointelligence* nationally and internationally. It unites over 40 institutions from science and industry [8].

Today, *biointelligence* describes the targeted integration of biological processes, structures, and ways of thinking into technical applications. This interdisciplinary approach promises not only efficiency gains but also a profound transformation of technical systems toward greater adaptability and resource conservation. *Biointelligence* is more than just using biological components – it stands for an intelligent interplay between technology and biology. It can be described as the systematic use of biological principles to solve technical challenges, transferring properties such as self-organization, learning ability, and energy efficiency to technical systems. Against this background, the question arises: What role does analytics play in biointelligent systems? And how must analytical methods and sensor technology evolve to meet these demands?

Biointelligent systems are highly complex, dynamic, and interactive. They require a new generation of analytical methods that not only collect data but also understand, interpret it, and can respond in real time. This is where the concept of Analytics 5.0 comes in – a new development stage of analytical sciences, evolving classical analytics toward intelligent, networked, and sustainable systems. Analytics 5.0, in the context of human-centered Industry 5.0, describes analytics that are: (1) *System-integrated*—Sensors are part of the biointelligent system, not just external observers. (2) *Adaptive*—Measurement systems dynamically respond to environmental conditions and process flows. (3) *Networked*—Data flows are bidirectional – sensors provide information and receive control impulses. (4) *AI-supported*—Artificial intelligence enables pattern recognition, forecasting, and autonomous decisions. (5) *Sustainable*—Resource-conserving measurement principles and energy-efficient sensor technology are key requirements [9–11]. This new form of analytics is not just a monitoring tool but an active component of biointelligent processes. It enables feedback loops, supports the self-organization of technical systems, and contributes to optimizing biological-technical interactions.

Sensor technology forms the basis for implementing AI applications in production. Modern sensor networks enable predictive maintenance, human–robot collaboration, and real-time monitoring of complex processes. AI-supported systems like Edge AI or digital twins analyze sensor data locally and enable fast, adaptive decisions [11]. A central element of Analytics 5.0 is the *FAIR principle* for scientific data: (1) *Findable* – data must be discoverable (e.g., via metadata and identifiers), (2) *Accessible* – data must be accessible (e.g., via standardized interfaces), (3) *Interoperable* – data must be compatible with other systems and formats, (4) *Reusable* – data must be reusable (e.g., through clear licensing and documentation) [12]. The FAIR principles were first formulated in 2016 and are now considered the standard for sustainable research data management; these are especially relevant for biointelligent systems that generate and process large amounts of complex data [13]. FAIR-compliant data infrastructures not only enable better scientific reproducibility but also integration into AI-supported decision-making processes. Examples of Analytics 5.0 in biointelligent applications include:

- Bioreactor control with learning sensors that analyze and adaptively regulate metabolic processes in real time.
- Organ-on-a-chip systems where microfluidic sensors monitor and control biological reactions.
- Agricultural systems where plants “communicate” with their environment via sensors and autonomously regulate irrigation or nutrient supply.
- Medical technology, e.g., implantable sensors that continuously record biological parameters and trigger therapeutic actions.

*Biosensors* are a key technology of biointelligent systems and play a central role as interfaces between biological and technical components [14–17]. They enable the detection of biological signals that can be translated into technical control processes – for example, for bioprocess regulation, medical diagnostics, or environmental monitoring. In many cases, they are considered biointelligent themselves [17–19]. Integrating biosensor technology into biointelligent systems also requires structured data management as defined by the FAIR principles [12].

The performance of biosensors largely depends on the quality of recognition structures. An extremely large number exists and should not be discussed at this point [20]. Second, detection principles also play a major role. Sensors can be categorized based on the underlying physical effect into the following main groups: (1) Electrochemical sensors – detection via electrochemical reactions (e.g., pH, redox potential, ion concentration) [21, 22]. (2) Thermal sensors – measure temperature changes (e.g., thermoelements, thermistors, pyrometers) [23, 24]. (3) Mechanical sensors – piezoelectric sensors, surface acoustic waves (SAW), quartz crystal microbalances (QCM) [25–28]. (4) Optical sensors – divided into label-based and direct optical sensors [29, 30]. Optical sensors were the focus of research in my group, so they will be discussed in more detail and it will be demonstrated how they developed into biointelligent systems. Optical label-based methods offer excellent detection limits but are often critical in terms of biocompatibility and susceptible to photochemical changes. Label-free methods allow direct detection without analyte modification but can be disturbed by nonspecific interactions. Direct optical detection methods can be divided into refractometric and reflectometric methods [31]. Refractometric sensors are based on changes in the refractive index and are particularly temperature-sensitive. Among refractometric methods, the most well-known is Surface Plasmon Resonance (SPR) (https://www.cytivalifesciences.com/en/us/about-us/our-brands/biacore), but waveguide-based systems, Mach–Zehnder or Young arrangements, ring resonators, and grating couplers are also common [31]. They are widely used and well commercialized, often using evanescent fields, which decay exponentially into the sample – unfavorable for cell measurements [31, 32]. Reflectometry of polarized light on thin layers was early used to characterize semiconductor surfaces [33]. Today, ellipsometry is essential in semiconductor industry processes for characterizing wafers for highly complex electronic components [34]. Early on, the potential of ellipsometry for characterizing biological layers was recognized [34]. Simplified experimental forms such as white-light interference (Fabry–Perot) can be used to observe changes in thin layers or deposits on such thin layers. Here, too, the product of the refractive index and the physical layer thickness is observed. However, in many applications, it has been shown that the temperature dependence of the refractive index plays no role and the signal does not decay exponentially as in the evanescent field-based sensors. This Reflectometric Interference Spectroscopy (RIfS) has been a focus for decades in our research team. It was introduced as a combination of classical interference principles with modern requirements for label-free, sensitive, and time-resolved detection methods in bioanalytics [35]. For use in measurements in the cavities of microtiter plates, it was modified and commercialized as Bio-Layer Interferometry (BLI) and is now sold for binding kinetics (https://www.sartorius.com/en/products/biolayer-interferometry).

Because of our many years of experience with RIfS and the development we have achieved, this method will be used as a model example for the development of a potential “biointelligent” transduction system. It can demonstrate necessary adjustments to real-world analytical challenges, steps to overcome general drawbacks of optical detection systems, and the development process to bring biosensors to biointelligent systems. Reflectometry can serve as an example of how optical sensors have evolved from single-point devices to imaging systems, the latter being particularly interesting for observing cell studies or complex biological processes. This will be illustrated using selected applications and demonstrate the potential benefits in the field of *biointelligence*. White light interferometry (RIfS) has been successfully used from the beginning in numerous applications, such as the quantification of pesticides in the environment [36] or for medical diagnostics in therapeutic drug monitoring [37]. RIfS has also been used as a chemosensor [38]. However, detection was not selective, and therefore the sensor had to be coupled with chemometrics [39]. Early applications in observing biological processes can be found in fermentation control [40], a field where *biointelligence* plays a role today. The biosensor is used to determine the optimal endpoint of fermentation before metabolites are produced. Since white light interferometry provides depth-independent information from layers without exponentially decaying signals as in evanescent techniques, cell behavior can be observed over time, such as the spreading process [41].

To simplify the measurement technique, the measurement white light spectrum was replaced by an arrangement with multiple colored LEDs [42]. Approaches to parallelization were also considered as precursors to measuring a sensor array, where multiple spots on the transducer were measured in parallel using an optical multiplexer with light guides. Chemometrics also played a role in the evaluation [43]. The previously used setups were discussed and compared [44].

For observing a two-dimensional array, as required for screening, a camera was used to capture the transducer surface instead of a fiber bundle approach. This was realized with sequential illumination at selected wavelengths rather than spectral exposure [45]. Influenced by user requirements at the time, the microtiter plate format was chosen as the surface. A color filter wheel generated individual illumination wavelengths per minute, and images of binding events in the microtiter plate cavities were recorded. A label-free high-throughput screening of thrombin inhibitors was the first application [46], followed by label-free parallel screening of combinatorial triazine libraries [47]. However, the format limited miniaturization, and the filter wheel technology was too complex. Therefore, an imaging RIfS was developed that continued the single-wavelength development and reduced the transducer to about one square centimeter, without cavities but with spots—up to 1000 per square centimeter [48]. This approach has the advantage that instead of cavities, the flat surface of the transducer in a flow cell can be used. This expands the possibilities for using different assays and allows for a defined evaluation of the binding kinetics. Numerous libraries were screened with it [49]. A modification was the use of polarized radiation for characterizing autoimmune diseases, which are characterized by the presence of autoantibodies in the serum of affected patients [50]. This imaging technology was commercialized under the name SCORE (single color reflectometry) [51]. More recently, a modified platform has been used to study initial cell adhesion to different extracellular matrix protein–coated surfaces [52].

SCORE as a single color reflectometry is a perfectly suited methodology for studying the entire cell adsorption process, including morphological changes. Artificial intelligence methods are also used in binding studies. Single-wavelength interference is currently being applied in an EU project called *BioPros* [53], a typical project dedicated to biointelligence. (a) Label-free detection: RIfS enables the observation of binding events without fluorescence or radioactivity modelling—a key criterion for *BioPros*. (b) Real-time measurement: RifS’s ability to determine kinetic parameters such as association and dissociation rates is essential for characterizing biomolecules. (c) Miniaturization and parallelization: Imaging RifS and 1–λ reflectometry enable simultaneous analysis of multiple samples—ideal for high-throughput screening, as pursued in *BioPros *.

At the beginning of the lecture, the question was asked: “Can the results of decades of sensor research contribute to *biointelligence*?” Looking at the development and present applications of optical sensors, we find the following answers to the question and find their significant contributions to the intentions of Analytics 5.0:

- *Sensors act as a bridge between biology and digitization*

Sensors capture biological, chemical, and physical processes in real time. They enable the transformation of analog biological signals into digital data, which can be used for AI-based analyses and modeling. Thus, they form the basis for digital twins.

- *Advances in optical sensing help*

The development of Reflectometric Interference Spectroscopy (RifS) exemplifies how sensing has evolved from simple white light systems to high-precision imaging techniques. Imaging RifS allows spatially and temporally resolved measurements, e.g., of cell adhesion, protein binding, or drug effects.

- *Applications with societal relevance emerge*
  1. *Therapeutic Drug Monitoring (TDM)*: Sensors enable individualized dosing of medications, e.g., for antidepressants or immunosuppressants.
  2. *Fermentation control*: Real-time monitoring of production processes in biotechnology.
  3. *Autoimmunity diagnostics*: Detection of disease-relevant antibodies without labelling.
  4. *Cell-based sensing*: Analysis of cell responses to stimuli—important for immunology and drug development.

In conclusion: Sensors are not just tools, but active components of biointelligent systems. They are the core component of *biointelligence.* They are coupled with AI to recognize patterns, make predictions, and support decisions. They are part of digital twins that model and optimize biological processes. Finally, sensors support Analytics 5.0, as they include human-centered, ethically reflected, and AI-supported analysis. Sensors support the goal of *biointelligence*: how to use technology meaningfully and responsibly in the field of life sciences and medicine.

## Declarations

1. Günter Gauglitz was a member of the International Advisory Board of this journal but not involved in the peer review of this contribution.

2. No data, text, or theories by others are presented as one’s own.

3. An attempt was made to cite references for all statements
